# Long Noncoding RNA MIR4697HG Promotes Cell Growth and Metastasis in Human Ovarian Cancer

**DOI:** 10.1155/2017/8267863

**Published:** 2017-01-10

**Authors:** Li-qian Zhang, Su-qing Yang, Ying Wang, Qiao Fang, Xian-jun Chen, Hong-sheng Lu, Ling-ping Zhao

**Affiliations:** ^1^Gynecology Department, Taizhou Central Hospital, Zhejiang 318000, China; ^2^Clinical Laboratory, Taizhou Central Hospital, Zhejiang 318000, China; ^3^Pathology Department, Taizhou Central Hospital, Zhejiang 318000, China

## Abstract

Ovarian cancer is one of the three most common gynecological malignant tumors worldwide. The prognosis of patients suffering from this malignancy remains poor because of limited therapeutic strategies. Herein, we investigated the role of a long noncoding RNA named MIR4697 host gene (MIR4697HG) in the cell growth and metastasis of ovarian cancer. Results showed that the transcriptional level of MIR4697HG in cancerous tissues increased twofold compared with that in adjacent noncancerous tissues. MIR4697HG was differentially expressed in ovarian cancer cell lines, with the highest levels in OVCAR3 and SKOV3 cells. MIR4697HG knockdown by specific shRNA significantly inhibited cell proliferation and colony formation in both OVCAR3 and SKOC3 cells. Consistently, in a xenograft model of ovarian cancer, MIR4697HG depletion also significantly restricted tumor volumes and weights. Furthermore, MIR4697HG knockdown inhibited cell migration and invasion capacities. Invasion ability was inhibited by 58% in SKOV3 cells and 40% in OVCAR3 cells, and migration ability was inhibited by 73% in SKOV3 cells and 62% in OVCAR3 cells after MIR4697HG knockdown. MIR4697HG knockdown also caused a decrease in matrix metalloprotease-9, phosphorylated ERK, and phosphorylated AKT. These data suggested that MIR4697HG promoted ovarian cancer growth and metastasis. The aggressive role of MIR4697HG in ovarian cancer may be related to the ERK and AKT signaling pathways.

## 1. Introduction

Ovarian cancer is one of the three most common gynecological malignant tumors and the third most common cancer in females worldwide. According to recent statistics, 22 280 new cases of ovarian cancer have emerged in the United States, among which 15 500 are estimated to die from this malignancy [1]. Currently, ovarian cancer patients have three major therapeutic options, namely, surgery, chemotherapy, and radiotherapy. Unfortunately, most patients relapse after surgery or develop resistance to chemotherapy drugs [2]. The prognosis of patients suffering from ovarian cancer remains poor because of limited therapeutic strategies and late diagnosis. Over 70% patients are estimated to be diagnosed at an advanced stage [1], and only approximately 30% of patients have a 5-year survival rate [3]. Hence, identifying novel targets is urgent for the early diagnosis and treatment of ovarian cancer.

Long noncoding RNAs (lncRNAs) widely exist in the nucleus and cytoplasm of eukaryotic cells. These RNAs are nonprotein coding and longer than 200 nucleotides [4, 5]. With the continuous improvement of research methods, lncRNAs have recently undergone a rapid expansion of research and discovery. An increasing number of studies have concluded that lncRNAs are closely associated with tumor development and progression [6]. In particular, several lncRNAs have elicited the interest of scientists and clinicians because of their specific roles in ovarian cancer. These lncRNAs include H19 [7–9], LSINCT5 [10], XIST [11, 12], MALAT-1 [13, 14], and ANRIL [15]. They been shown to be associated with various biological activities in ovarian cancer, including cell growth [10, 13], metastasis [13, 16], cell senescence [15], cell apoptosis [14, 15], and multidrug resistance [3, 17]. All these studies indicate that lncRNAs may play critical roles in the development and progression of ovarian cancer.

Recently, lncRNA MIR4697 host gene (MIR4697HG) has been identified to be a key competing endogenous RNA (ceRNA) for miRNA-mRNA in lung adenocarcinoma [18]. Using RNA-seq and miRNA-seq techniques, MIR4697HG and two other lncRNAs have been identified to be differentially expressed in lung adenocarcinoma and associated with clinical features [18]. However, the detailed role of MIR4697HG in lung cancer and other solid tumors remains largely unknown. The present study aimed to investigate the expression profile and functional role of MIR4697HG in ovarian cancer and thus be the first to unravel the critical role of a novel lncRNA (MIR4697HG) in ovarian cancer.

## 2. Materials and Methods

### 2.1. Reagents, Cell Lines, and Cell Culture

Specific shRNA against MIR4697HG (5′-GTGAGAATCACTCTCCCATGGATCAGTGTGGGCCCTGTCCCTCTTCCCTTTTT-3′) was designed and synthesized by Invitrogen (Shanghai, China). A negative control shRNA was synchronously synthesized. Primary antibodies against MMP-9 and GAPDH were commercially purchased from Abcam (Hong Kong, China). Antibodies against ERK, phosphorylated EKR (p-ERK), AKT, and phosphorylated AKT (p-AKT) were obtained from Cell Signaling Co. (NY, USA). Four ovarian cancer cell lines, CoC1, CaoV-3, OVCAR3, and SKOV3, were purchased from the American Type Culture Collection (ATCC, USA) and maintained in Dulbecco's modified Eagle's medium (DMEM) (Invitrogen, CA, USA) supplemented with 10% fetal bovine serum (FBS, Invitrogen) and 100 U/mL penicillin/streptomycin (Sigma, St. Louis, MO, USA). Cells were incubated at 37°C in humidified atmosphere of 5% CO_2_. Cell culture medium was refreshed every two days.

### 2.2. Human Tissues and Ethical Statements

Fifteen cases of ovarian cancer tissues and their adjacent noncancerous tissues were collected from patients who underwent ovariectomy at the Department of Gynecology, Taizhou Central Hospital. These patients have received no chemotherapy or radiotherapy prior to surgical resection. All cases were diagnosed with ovarian cancer by two independent pathologists without any controversial. Written consent form was obtained from each patient. Protocols for using these samples for research purposes were approved by an Institutional Review Board at Taizhou Central Hospital.

### 2.3. Western Blot Analysis

Total proteins were extracted from transfected cells. Extracted proteins were quantified using a BCA kit (Beyotime, Nantong, China). An equal amount of 50 ng proteins were then loaded to a 12% SDS-PAGE gel, followed by being transferred onto PVDF membranes (pore size = 0.45 *μ*m) (Millipore, Billerica, MA, USA). After blocking in 5% skim milk in Tris-based saline-Tween 20 (TBST) for 60 min at room temperature, membranes were incubated with corresponding primary antibodies overnight at 4°C. The membranes were thereafter washed with TBST for three times and further incubated with a secondary antibody for 1 hour at room temperature. Enhanced chemiluminescence (ECL) solution was used to develop the immunoreactivity. Glyceraldehyde 3-phosphate dehydrogenase (GAPDH) was synchronously detected as a loading control.

### 2.4. RNA Isolation and Quantitative Real-Time Polymerase Chain Reaction (qRT-PCR)

Total cellular RNAs were isolated from human tissues and ovarian cancer cell lines using TRIzol reagent (Takara Bio, Inc., Shiga, Japan) and then reversely transcribed into cDNA using PrimeScript RT master Mix (Takara) based on the manufacturer's instructions. qPCR was performed to determine the transcription level of MIR4697HG and GAPDH mRNA using a SYBR GREEN MIX kit from Promega (Madison, WI, USA) according to the manufacturer's protocols. The transcription level of MIR4697HG was then normalized to that of GAPDH.

### 2.5. Cell Viability and Colony Formation Assays

Prior to experiments, SKOV3 cells and OCVAR3 cells that were stably depleted of MIR4697HG were constructed. To assess proliferation of ovarian cancer SKOV3 cells and OVCAR3 cells, 4,500 cells that were treated with specific shRNA (shRNA group) or negative control shRNA (NC group) were seeded per well in a 96-well plate and cell growth was assessed using the Cell Counting Kit-8 (CCK-8) for consecutive 5 days according to the manufacturer's instructions. For each monitored time point, an aliquot of 10 *μ*L CCK-8 was added to each well. Cells were then incubated at 37°C for another 2 hours. Cell viability was then determined by detecting the absorbance at 450 nm for each group. To determine the long-term effect of MIR4697HG knockdown on cell growth, colony formation assays were performed. Briefly, 500 cells were plated per well in 6-well plates. Cells were allowed to grow for 2 weeks when colonies formed were stained with crystal violet. The stained colonies were then photographed and manually counted.

### 2.6. Transwell Migration and Invasion Assays

Transwell chambers with 8 *μ*m pores were obtained from Corning (Corning, NY, USA). After transfection with NC or specific shRNA, SKOV3 cells and OCVAR3 cells were harvested, resuspended in 100 *μ*L of FBS-free DMEM at an initial concentration of 1 × 10^6^ cells. Cells were then seeded into the upper chambers of a 24-well plate. The lower chambers were filled with 600 *μ*L DMEM containing 10% FBS. Cells were then incubated for another 12 hours. At the end of the experiment, cells that transmigrated to the underneath surface of the transwell membrane were fixed with methanol, stained with crystal violet, and then photographed under a light microscope at 100x magnification. For each group, five fields were randomly selected. Transmigrated cells in the five fields were counted manually. Cell numbers of the five visual fields were then averaged. For the transwell invasion assays, the membrane was precoated with 50 *μ*L of Matrigel (1 : 3 mixed with PBS; BD Biosciences, Heidelberg, Germany) and proceeded the same as described above.

### 2.7. A Xenograft Model of Ovarian Cancer

A xenograft model of ovarian cancer was established using SKOV3 cells with MIR4697HG depletion (shRNA group) or not (NC group). Briefly, 6-week-old athymic nude mice were randomly assigned to two groups (*n* = 5 for each group). For each group of mice, transfected SKOV3 cells (2 × 10^4^) were injected into the right flank. Mice were then monitored for the growth of tumors. Tumor length (*L*) and width (*W*) were measured twice a week. And tumor volume (TV) was calculated as TV = *L* × *W*^2^/2. By day 28 after inoculation, all mice were sacrificed and tumors were dissected. The dissected tumors were weighed and photographed. All efforts were made to minimize suffering. Protocols for animal experiments were approved by the Ethics Committee from Taizhou Central Hospital.

### 2.8. Statistical Analysis

All data were expressed as mean ± standard deviation (SD). Differences between groups were assessed using Student's *t*-test. A difference with the *p* value less than 0.05 was considered statistically significant.

## 3. Results

### 3.1. MIR4697HG Is Upregulated in Ovarian Cancer Tissues and Differentially Expressed in Ovarian Cancer Cell Lines

**Figure 1:**
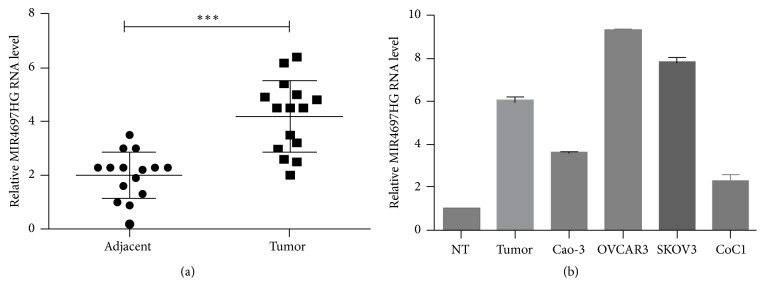
MIR4697HG is upregulated in ovarian cancer tissues and differentially expressed in ovarian cancer cell lines. (a) qRT-PCR analysis of the MIR4697HG level in 15 cases of ovarian cancer tissues and their adjacent noncancerous tissues. ^*∗∗∗*^*p* < 0.001. (b) The relative MIR4697HG level in normal ovary tissues (NT), ovarian cancer tissues, and four ovarian cancer cell lines (Caov-3, OVCAR3, SKOV3, and CoC1) were examined.

Initially, the expression profile of MIR4697HG in ovarian cancer was examined. Results showed that the relative transcriptional level of MIR4697HG in ovarian cancerous tissues was twice that in matched adjacent noncancerous tissues (Figure 1(a)). Moreover, the relative MIR4697HG level in ovarian cancer tissues was sixfold compared with that in normal ovary tissues (Figure 1(b)). Thus, MIR4697HG was upregulated in ovarian cancer. Interestingly, MIR4697HG was differentially expressed in the four ovarian cancer cell lines. OVCAR3 cells exhibited the highest MIR4697 level, followed by SKOV3 cells. Caov-3 and CoC1 expressed the least level of MIR4697HG (Figure 1(b)).

### 3.2. MIR4697HG Knockdown Inhibited Cell Proliferation and Colony Formation in SKOV3 and OVCAR3 Cells

**Figure 2:**
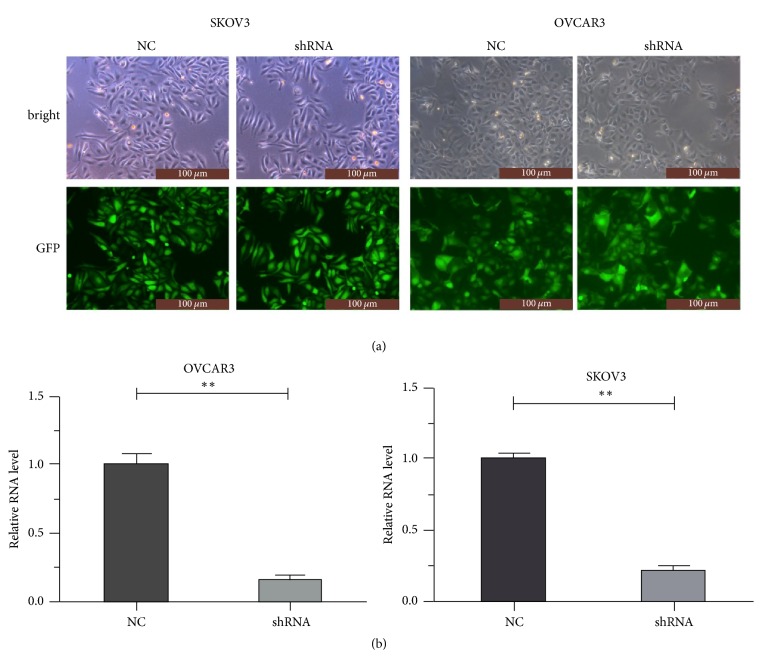
Successful depletion of MIR4697HG by a specific shRNA. (a) After transfection of negative control (NC) or specific shRNA into cells, cells were photographed under a bright vision and a fluorescence microscopy. Ninety percent cells were shown to express green fluorescence protein (GFP). (b) The specific shRNA decreased the MIR4697HG level by up to 72% in SKOV3 cells and 84% in OVCAR3 cells. ^*∗∗*^*p* < 0.01.

**Figure 3:**
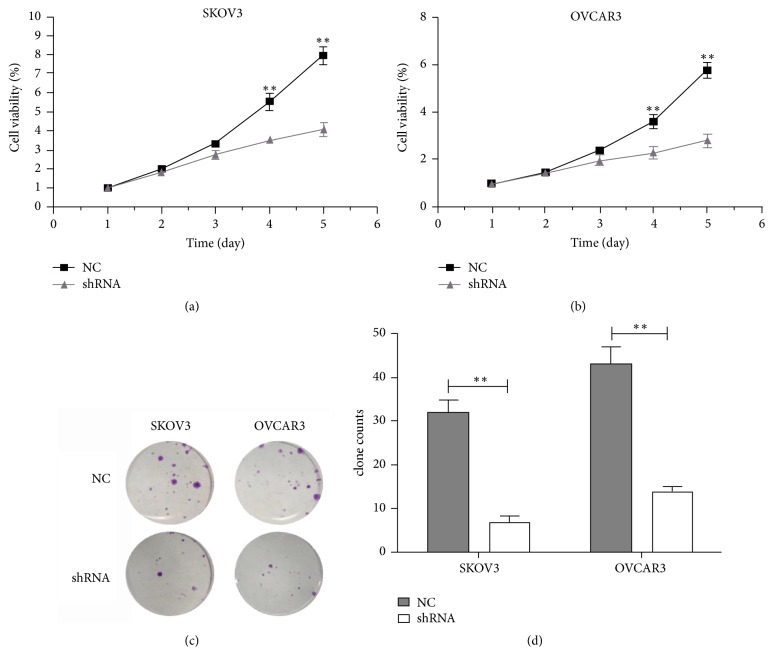
Knockdown of MIR4697HG inhibits cell viability and clonogenic potential in SKOV3 cells and OVCAR3 cells. (a) SKOV3 cells with (shRNA group) or without (NC group) specific shRNA transfections were subject to cell viability assessment for consecutive 5 days. (b) OVCAR3 cells with (shRNA group) or without (NC group) specific shRNA transfections were subject to cell viability assessment for consecutive 5 days. (c) Both SKOV3 cells and OVCAR3 cells with (shRNA group) or without (NC group) specific shRNA transfections were subject to colony formation assays. After crystal violet staining, colonies were visually less observed in MIR4697HG-depleted SKOV3 cells and OVCAR3 cells. (d) Counting of colonies showed that colony numbers were significantly decreased by 75% in MIR4697HG-depleted SKOV3 cells and 66% in MIR4697HG-depleted OVCAR3 cells. ^*∗∗*^*p* < 0.01.

A specific shRNA against MIR4697HG was used to stably deplete MIR4697HG expression. After transfection of negative control (NC) or specific shRNA into cells, 90% cells were expressing green fluorescence protein, indicating successful transfection efficiency (Figure 2(a)). Specific shRNA also decreased the MIR4697HG level by up to 72% in SKOV3 cells and 84% in OVCAR3 cells (Figure 2(b)). Upon the successful MIR4697HG depletion in SKOV3 and OVCAR3 cells, cell proliferation and clonogenic potential were assessed. Cell viability assays revealed that MIR4697HG knockdown in SKOV3 cells significantly decreased cell proliferative rates since day 4. By day 5, proliferative rate was inhibited by approximately 62% after MIR4697HG knockdown (Figure 3(a)). Similarly, cell proliferation was significantly inhibited by approximately 64% in OVCAR3 cells with MIR4697HG depletion (Figure 3(b)). In colony formation assays, colonies were visually less observed after MIR4697HG knockdown in both SKOV3 cells and OVCAR3 cells (Figure 3(c)). Counting of colonies showed that colony numbers significantly decreased by 75% in MIR4697HG-depleted SKOV3 cells and 66% in MIR4697HG-depleted OVCAR3 cells (Figure 3(d)). These data suggested that MIR4697HG knockdown inhibited cell proliferation and clonogenic potential in ovarian cancer cells.

### 3.3. MIR4697HG Knockdown Inhibited Tumor Growth In Vivo

**Figure 4:**
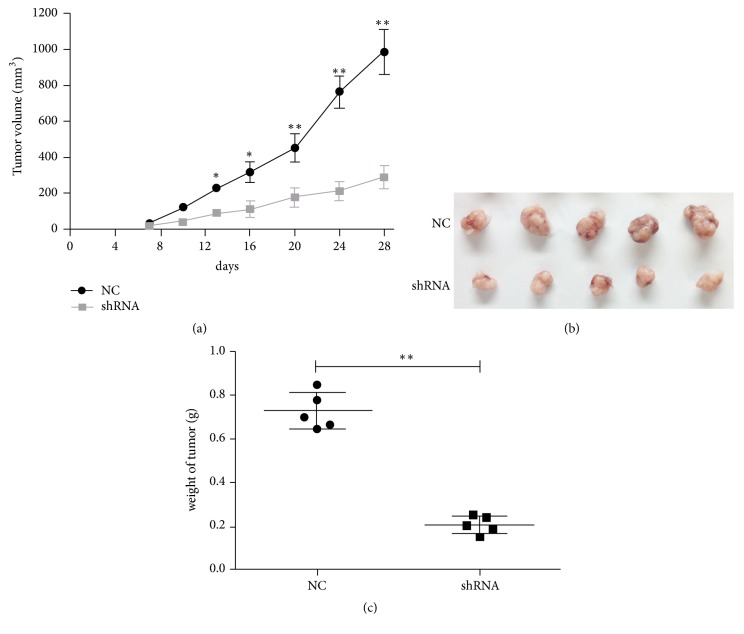
Knockdown of MIR4697HG inhibits tumor growth in vivo. (a) A xenograft model of ovarian cancer was established using SKOV3 cells with (shRNA group) or without (NC group) MIR4697HG depletion. Tumor size was periodically monitored. (b) Tumors were dissected from each mouse and shown for each group. (c) Dissected tumors were weighed for each group. It was shown that the average tumor weight was significantly less in the shRNA group, accounting for only 71% of that in the control group. ^*∗*^*p* < 0.05; ^*∗∗*^*p* < 0.01.

We established a xenograft model of ovarian cancer using SKOV3 cells with (shRNA group) or without (NC group) MIR4697HG depletion. Tumor size was periodically monitored. The average tumor volume in the shRNA group was smaller than that in the control group since day 13 after inoculation. By day 28, tumor volume in the shRNA group was only 77% of that in the control group (Figure 4(a)). After dissection of all tumors from each mouse, tumor size was visually smaller in the shRNA group than in the control group (Figure 4(b)). Consistently, average tumor weight was significantly less in the shRNA group, accounting for only 71% of that in the control group (Figure 4(c)). These data confirmed that MIR4697HG knockdown inhibited tumor growth in vivo.

### 3.4. MIR4697HG Knockdown Inhibited Cell Migration and Invasion in SKOV3 Cells and OVCAR3 Cells

**Figure 5:**
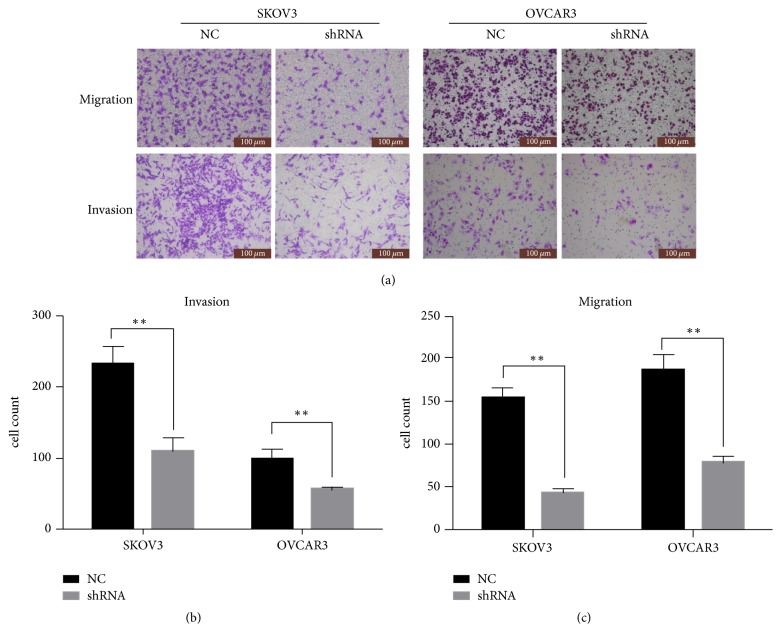
Knockdown of MIR4697HG inhibits cell migration and invasion in SKOV3 cells and OVCAR3 cells. (a) Both SKOV3 cells and OVCAR3 cells with (shRNA group) or without (NC group) specific shRNA transfections were subject to transwell migration and invasion assays. The transmigrated cells underneath the membrane were stained with crystal violet and photographed under a light microscopy. Five representative images were randomly selected for each group. (b) Invaded cells were counted in both SKOV3 cells and OVCAR3 cells with MIR4697HG knockdown (shRNA group) or not (NC group). Data were collected from five randomly selected fields and averaged for each group. (c) Migrated cells were counted in both SKOV3 cells and OVCAR3 cells with MIR4697HG knockdown (shRNA group) or not (NC group). Data were collected from five randomly selected fields and averaged for each group. ^*∗∗*^*p* < 0.01.

Effects of MIR4697HG knockdown on cell metastasis were subsequently assessed.  Figure 5(a) shows that, in transwell assays, migrated and invaded cells were visually inhibited after MIR4697HG knockdown in both SKOV3 cells and OVCAR3 cells (Figure 5(a)). Transmigrated cells were then counted. In the invasion assay, only approximately 100 MIR4697HG-depleted SKOV3 cells were found to invade through the Matrigel, in contrast to the almost 240 cells in the control SKOV3 cells (58% decrease). Similarly, 60 MIR4697HG-depleted OVCAR3 cells invaded through the Matrigel, which accounted for only 60% of control OVCAR3 cells (Figure 5(b)). In the migration assay, cell migration ability was found to be inhibited by 73% in SKOV3 cells and 62% in OVCAR3 cells after MIR4697HG knockdown (Figure 5(c)). All these observations suggested that MIR4697HG knockdown inhibited cell migration and invasion abilities in ovarian cancer cells.

### 3.5. MIR4697HG Knockdown Decreased the Levels of Matrix Metalloprotease-9 (MMP-9), Phosphorylated ERK (p-ERK), and Phosphorylated AKT (p-AKT) in SKOV3 Cells

**Figure 6:**
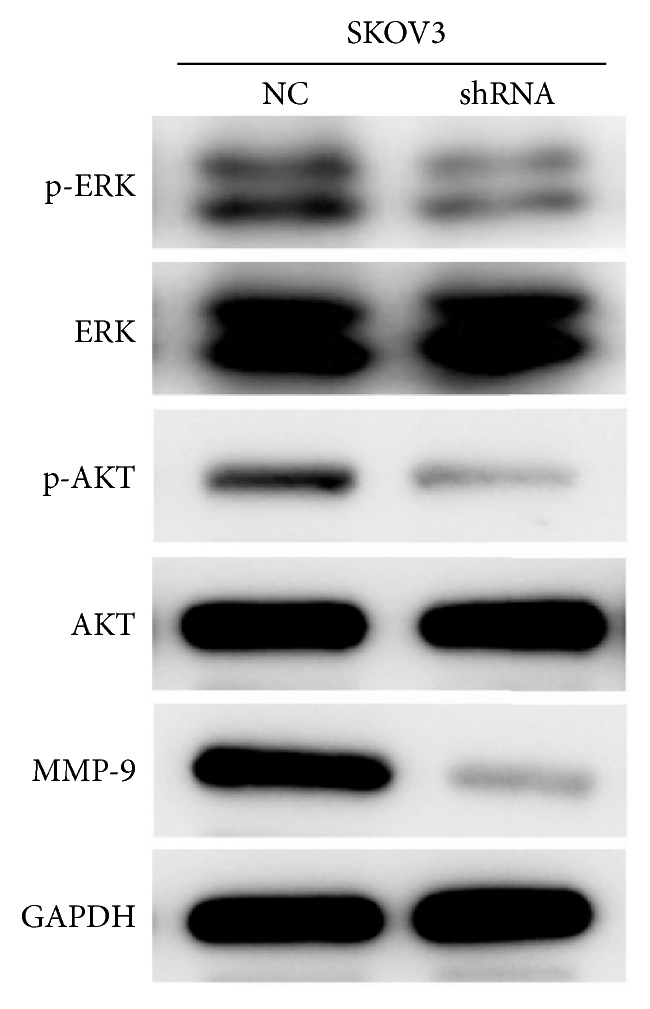
Knockdown of MIR4697HG decreased the levels of MMP-9, phosphorylated ERK, and AKT in SKOV3 cells. SKOV3 cells with (shRNA group) or without (NC group) MIR4697HG knockdown were subject to protein extraction. The extracted proteins were then assayed using western blot analysis of matrix metalloproteinase 9 (MMP-9), ERK (phosphorylated or not), and AKT (phosphorylated or not).

Western blot analysis further showed that, after MIR4697HG knockdown in SKOV3 cells, the protein level of MMP-9, a biomarker of tumor metastasis, significantly decreased. Interestingly, the total protein levels of ERK and AKT remained unchanged. However, the levels of p-ERK and p-AKT significantly decreased compared with their levels in control cells (Figure 6).

## 4. Discussion

Ovarian cancer is a great threat to female health worldwide. Approximately 30% of patients are estimated to have a 5-year survival rate [3, 19]. Such high mortality renders ovarian cancer the fifth entity accounting for 6% of all cancer-related deaths in females [1]. To increase the survival rate of patients, early diagnosis and timely medical intervention have been proposed [20]. Early detection of ovarian cancer at its localized stage can increase the 5-year survival rate to approximately 90% [20]. Hence, identifying novel targets that could serve as biomarkers for early diagnosis and treatment of ovarian cancer is urgent.

Researchers who are committed to the search for effective early ovarian cancer diagnostic markers have found that the development of ovarian cancer is associated with the aberrant expression of specific lncRNAs. The present study investigated the role of a newly identified lncRNA, that is, MIR4697HG, in cell growth and metastasis in ovarian cancer. MIR4697HG expression was significantly upregulated in ovarian cancer tissues. MIR4697HG depletion by specific shRNA slowed down proliferative rates in both SKOV3 cells and OVCAR3 cells. Clonogenic potential was consistently inhibited after MIR4697HG knockdown. A xenograft model of ovarian cancer further confirmed that MIR4697HG depletion inhibited tumor growth in vivo. All these data suggested the tumorigenic role of MIR4697HG in ovarian cancer. Interestingly, SKOV3 and OVCAR3 cells that were depleted of MIR4697HG exhibited less migration and invasion abilities. MMP-9, a marker for tumor distant metastasis, was also decreased by MIR4697HG knockdown in SKOV3 cells, which further confirmed MIR4697HG-mediated migration and invasion inhibition. Collectively, our data showed that MIR4697HG knockdown inhibited cell growth and metastasis in ovarian cancer.

Uncovering the mechanisms of MIR4697HG-mediated cell proliferation and metastasis in ovarian cancer is interesting. Currently, several mechanisms have been proposed to underlie the functional roles of lncRNAs. These complex mechanisms mainly include epigenetic modification through interaction with chromatin or methylation and ubiquitination, direct binding of transcription factors or heterologous DNAs to form multimeric transcription regulation complexes, and posttranscription regulation (splicing, transport, translation, and degradation of messenger RNA) [2, 21, 22]. As a novel lncRNA, MIR4697HG was initially identified to be a ceRNA serving as a molecular sponge for miRNAs and thus functionally liberating mRNA, ultimately leading to the upregulation of the aforementioned miRNA-targeted genes [18]. Thus, the lncRNA-miRNA-mRNA axis may be crucial to the role of MIR4697HG in human tumorigenesis. Our data further showed that after MIR4697HG knockdown in SKOV3 cells, the levels of p-ERK p-AKT significantly decreased, whereas their total protein levels remained unchanged. ERK/MAPK signaling and PI3K/AKT signaling are widely implicated in the promotion of cell growth and the suppression of apoptotic machinery after phosphorylase activation [23–25]. Activation of the ERK and AKT pathways induces cell growth and stimulates Bax-mediated proapoptosis [26]. Decreased p-ERK and p-AKT by MIR4697HG knockdown in turn suggested that MIR4697HG may regulate ERK and AKT signal pathways. In view of the ceRNA hypothesis, MIR4697HG may function as lncRNA sponge for specific miRNAs, in this way liberating key gene mRNAs that subsequently lead to the activation of the ERK and AKT signal pathways. If this scenario was true, identifying the specific miRNAs sponged by MIR4697HG in ovarian cancer would be very interesting. Taken together, the mechanisms of MIR4697HG-mediated cell proliferation and metastasis in ovarian cancer remain to be uncovered. More work is needed to test the ceRNA hypothesis in ovarian cancer.

In sum, we identified novel lncRNA MIR4697HG as a critical mediator of tumor growth and metastasis in ovarian cancer. MIR4697HG was significantly upregulated in ovarian cancer tissues. MIR4698HG knockdown significantly inhibited cell proliferation, clonogenic potential, and motility. To our knowledge, this study was the first to reveal the functional role and possible mechanisms of MIR4697HG in a solid tumor. Our data may provide evidence that MIR4697HG served as a significant biomarker for early ovarian cancer detection. Using specific shRNA or the developing novel targets against MIR4697HG may be promising strategies for the early treatment of this disease.
